# Therapeutic vaccines in HBV: lessons from HCV

**DOI:** 10.1007/s00430-014-0376-8

**Published:** 2015-01-09

**Authors:** Eleanor Barnes

**Affiliations:** 1 The Jenner Institute, The Oxford NIHR BRC and the Oxford Martin School, Oxford, UK; 2 The Peter Medawar Building for Pathogen Research, Oxford University, South Parks Rd, Oxford, OX1 3SY UK

**Keywords:** HBV, Immunotherapy, Adenoviral vectors, HCV, Checkpoint inhibitors

## Abstract

Currently, millions of people infected with hepatitis B virus (HBV) are committed to decades of treatment with anti-viral therapy to control viral replication. However, new tools for immunotherapy that include both viral vectors and molecular checkpoint inhibitors are now available. This has led to a resurgence of interest in new strategies to develop immunotherapeutic strategies with the aim of inducing HBeAg seroconversion—an end-point that has been associated with a decrease in the rates of disease progression. Ultimately, a true cure will involve the elimination of covalently closed circular DNA which presents a greater challenge for immunotherapy. In this manuscript, I describe the development of immunotherapeutic strategies for HBV that are approaching or currently in clinical studies, and draw on observations of T cell function in natural infection supported by recent animal studies that may lead to additional rational vaccine strategies using checkpoint inhibitors. I also draw on our recent experience in developing potent vaccines for HCV prophylaxis based on simian adenoviral and MVA vectors used in prime–boost strategies in both healthy volunteers and HCV infected patients. I have shown that the induction of T cell immune responses is markedly attenuated when administered to people with persistent HCV viremia. These studies and recently published animal studies using the woodchuck model suggest that potent vaccines based on DNA or adenoviral vectored vaccination represent a rational way forward. However, combining these with drugs to suppress viral replication, alongside checkpoint inhibitors may be required to induce long-term immune control.

## The goals for HBV immunotherapy

There has been a recent resurgence in interest to develop novel strategies for therapeutic vaccination against hepatitis B virus (HBV) within both academia and pharma. This has primarily been driven by progress in the treatment of HBV and changing therapeutic goals and new tools for immunotherapy that include both viral vector strategies and molecular checkpoint inhibitors. A number of new drugs—principally tenofovir and entecavir—that are safe and well tolerated with a high barrier to resistance have recently revolutionised the treatment of HBV [1–4]. However, with time it has been clear that the rates of HBV surface antigen (HBsAg) loss and HBV “e” antigen (HBeAg) seroconversion with these new therapies are very low so committing most patients to lifelong therapy [4, 5]. Furthermore, deploying lifelong therapies in resource-poor countries where the rates of HBV infection are the highest is rarely an option. In addition, there are emerging efforts to provide cure for HIV infection where some of the challenges parallel those found in HBV including persisting viral genomes and lifelong therapies [6]. Together, these observations have provided a new impetus to identify new strategies to cure HBV infection in patients currently receiving anti-viral therapy.

Hope that immunotherapy may be a successful strategy for HBV comes from two observations; firstly, that robust adaptive immune responses are associated with viral control during acute HBV infection disease [7–9] and secondly, that in chronic HBV disease, spontaneous viral control with suppression of HBV DNA is associated with immune activation as evidenced by HBeAg seroconversion and liver inflammation with a flare in liver alanine transaminases (ALT) [10]. This is in contrast to human immunodeficiency virus (HIV) where persistent infection is almost always established after primary disease and to hepatitis C virus (HCV) infection, where immune control associated with spontaneous viral control is established is exceedingly rare once chronic disease is established.

The goals of immunotherapy are therefore twofold: (1) To induce HBeAg seroconversion in those on therapy; this end-point has been associated with lower rates of disease progression to cirrhosis and hepatocellular carcinoma, an increase in the rates of HBsAg seroconversion, and improved survival rates [11], and (2) more ambitiously, to provide a complete cure that would require the elimination of HBV covalently closed circular (ccc) DNA and therefore disappearance of its major expression product HBsAg with or without anti-HBs seroconversion.

A number of strategies are available for HBV immunotherapy. Some of these rely on emerging molecular technologies and include T cell receptor gene transfer and therapeutic antibody conjugates. However, these are technically challenging, carry significant safety concerns and are unlikely to be applicable in resource-poor settings. These technologies are discussed in detail by Bertoletti et al. in this issue. This article will therefore focus on new technologies that are close to the clinic, in particular, strategies to enhance adaptive immune responses, specifically T cells, and how these may be successfully combined with oral therapies and other adjuvants.

## The rationale for HBV immunotherapeutic approaches

One of the key challenges for immunotherapy is the development of strategies that enhance adaptive T cell responses in the setting of chronic high-level antigen exposure. A hallmark of persistent infection with HBV is a markedly attenuated or “exhausted” T cell response; low in magnitude, narrowly focused, poorly proliferative, and producing low levels of interleukin (IL)-2 [12, 13]. Advancing our understanding of the underlying mechanism for T cell attenuation is important for the development of rational therapeutic strategies. HBV infection is characterised by the persistent production of very high quantities of subviral HBsAg particles, and a secreted form of HBV core antigen named “e” antigen (HBeAg) that exceeds by a thousand-fold or more the amount needed for assembly of complete HBV particles. However, the direct role of exposure to these antigens in attenuating T cell immunity is controversial. Since HBV polymerase is not secreted in this way and T cell responses to polymerase are also attenuated, it is unlikely that these antigens are wholly responsible for the exhausted T cell profile observed.

Certainly, there is mounting evidence that T cell priming in a tolerating liver environment is likely to be critical, in addition to the premature death of HBV-specific T cells in the liver through proapoptotic protein Bcl2-interacting mediator (Bim)-dependent mechanisms [14, 15]. Furthermore, HBV has been shown to induce the liver enzyme arginase that depletes hepatic arginine resulting in T cells that are poorly proliferative [16]. A unifying hypothesis then is that HBV-specific T cells may be driven to apoptosis through the interplay between (1) high dose antigen, (2) the high expression of inhibitory signals, and (3) lack of co-stimulatory molecules and other inhibitor mechanisms within the liver. Immunotherapeutic strategies should seek then to target these factors and to induce functional anti-viral T cells by vaccination.

### HBV viral suppression

Taking these factors in turn, the suppression of HBV viral replication may be readily achieved using current oral therapies. This would serve two purposes—to reduce the chance of immunopathology in the context of immunotherapeutic vaccination and possibly to facilitate the recovery of adaptive immune responses. Mouse models of lymphocytic choriomeningitis virus (LCMV) infection suggest that recovery of adaptive responses are best achieved in the setting of low viral load [17], and in support of this, there is good evidence that HBV viral load suppression with oral anti-viral therapy is sometimes associated with immune activation and HBeAg seroconversion [18]. More typically, however, in the absence of HBeAg seroconversion, viral suppression with oral therapies is associated with a transient, early, and minimal increase in adaptive immune responses only [19]. Theoretically, it is plausible that intermittent, low levels of viral replication are required to stimulate immune control of virus. It should be noted that whilst oral anti-virals reduce infectious virus through the inhibition of HBV reverse transcriptase, they have little effect on HBV antigen levels that are generated through transcription by RNA polymerase II, and no direct effect on HBV ccc DNA. Overall, since viral load suppression alone is generally insufficient to restore adaptive response, successful immunotherapy will need to be combined with additional strategies.

### Checkpoint inhibition

The high expression of inhibitory molecules on HBV-specific T cells is well described and provides some interesting molecular targets for additional immunotherapeutic strategies including programmed cell death protein 1 (PD-1), cytotoxic T lymphocyte antigen 4 (CTLA-4), 2B4, T cell membrane protein 3 (Tim-3), Lymphocyte-activation gene 3 (LAG-3), and their ligands—some of which are currently available for clinical use or in late stage development.

To date, these checkpoint inhibitors have largely been given to patients with advanced malignancy. CTLA-4 has been linked to Bim expression, and the blocking of CTLA-4 in vitro has been linked to the expansion of HBV-specific T cells. HBV viral load suppression with anti-viral therapy is not associated with a reduction in CTLA-4 or Bim levels on human HBV-specific T cells [15]. CTLA-4 inhibitors are currently available in the clinic; ipilimumab (BMS) has been used successfully to treat patients with a range of malignancies including melanoma, prostate, and renal cancer, and was approved in 2011 by US regulators for the treatment of melanoma. However, this monoclonal antibody has been associated with immune-related side effects that include reversible colitis, rashes, and hepatitis [20]. In practice, it is unlikely that CTLA-4 checkpoint inhibitors will move into clinical studies of HBV in the near future since the safety profile of these are unacceptable in HBV patients who are otherwise well.

PD-1 and PD-1 ligand inhibitors are also in advanced development including three monoclonal antibodies, and three other drugs (reviewed in [21]) now in phase-I to phase III clinical trials. Therapeutic anti-PD-1 antibodies have been assessed in three chimpanzees and in humans with chronic HCV infection, but not in human studies of HBV infection. In one study of three chimpanzees chronically infected with HCV, pre-existing intrahepatic T cell responses and CD4+ and CD8+ HCV-specific T cells were restored, associated with a transient but substantial decline in HCV viral load showing for the first time that reversal of T cell tolerance induced by hepatotropic viruses is possible [22]. The anti-PD-1 monoclonal immunoglobulin G4 (Nivolumab; BMS-936558) was subsequently assessed in 54 patients with chronic HCV infection; five achieved a >0.5 log decline in HCV viral load, two achieved a viral load decline below the level of detection, and one patient became HCV undetectable. Immune-related side effects were seen in six patients, and in one patient, there was a grade 4 elevation in ALT associated with viral decline [23]. Overall, grade 3 and grade 4 adverse events have been seen in around 15 % of patients including deaths from pneumonitis in oncology patients treated with nivolumab [24].

In spite of the significant adverse event profile, it is likely that anti-PD-1 immunotherapeutic strategies will be assessed in HBV phase-I studies in the short term. Clinical experience in the use of anti-PD-1 strategies is growing, and overall the side effects are thought to be manageable. Furthermore, recent animal studies using a woodchuck model have shown promise for immunotherapeutic strategies targeting the PD-1 pathway as an adjunct to vaccination; PD-1 blockade in combination with both entecavir and DNA vaccination increased virus-specific T cell function associated with anti-woodchuck hepatitis surface antibodies and viral eradication in some animals [25]. LAG-3 inhibitors are currently in phase-I clinical studies of solid tumours (ClinicalTrials.gov NCT01968109), whilst TIM-3 inhibitors are in advanced pre-clinical development.

### Lessons from studies of HBV immunotherapy

Previous attempts at therapeutic HBV vaccination have induced very low levels of T cells with minimal effects on viral suppression or HBV seroconversion events. Early efforts utilised HBsAg protein vaccines primarily inducing antibodies with minimal effects in established infection [26–28]. HBV single and multi-epitope string vaccines have induced little immunogenicity, and these approaches are unlikely to give sufficient coverage across HBV genotypes, or between people of distinct HLA types [29].

DNA vaccinations that encode small (S) and middle (preS2 and S) envelope proteins have also had limited success; these have induced anti-preS2 antibodies and HBeAg seroconversion in the minority of HBV vaccines (2/10) associated with weak and transient T cell responses [30, 31]. However, repeated vaccinations (*n* = 12) with DNA encoding S, preS1/S2, core, polymerase and X proteins with genetically adjuvanted IL-12 and oral anti-virals induced a multi-specific T cell response, sustained viral suppression, and HBeAg seroconversion in 6/12 patients [32, 33]. A recent pilot study of 36 patients with chronic HBV infection used electroporated DNA encoding HBV middle S protein with an IL-12/IFN gamma fusion protein given with lamivudine. This was associated with the generation of T cell immune responses with a reduction in HBV viral load in some people [33]. However, DNA vaccine strategies that require painful electroporation or multiple injections to generate significant immune responses are unlikely to be widely clinically applicable. For these reasons, heterologous prime/boost vaccination strategies using viral vectors have been assessed as an alternative strategy. DNA prime/MVA boost vaccine encoding S, preS1/S2 showed promise in a chimpanzee study [34]. However, when the same vaccine was assessed in a clinical study in Gambia given with or without lamivudine, there was no T cell induction or reduction in viremia [35]. The reasons for this may relate to the fact that the vaccine contained S and preS immunogens only. However, strategies that reduce HBV viral load with drug therapy before DNA prime/Ad boost encoding HBV core has shown real promise in the HBV woodchuck model; this strategy was able to induce a robust CD4+ and CD8+ T cell response associated with control of viremia and HBsAg seroconversion [36]. Transgene, a French biopharmaceutical company developing viral vectored approaches for the treatment of infectious disease, is planning to assess non-replicative Ad5 vectors (TG1050) encoding multiple HBV antigens (Core, Polymerase and Envelope) from genotype D HBV in China in late 2014 (press-release July 2014).

### Innate immune stimulation

One of the advantages of a virally vectored vaccine is that the vectors themselves will induce innate immune responses. This may be important given the observation that innate immune stimulation alone may be associated with HBV viral control though mechanisms that are currently not clear. For example, the administration of Granulocyte–macrophage colony-stimulating factor (GM–CSF) has been associated with HBV DNA and/or HBeAg loss in up to 60 % of treated patients [37, 38]. Furthermore, a comparison of two consecutive studies assessing an antigen–antibody (HBsAg–HBIG) immunogenic complex therapeutic vaccine candidate with alum as adjuvant and alum alone as placebo suggested that increasing the frequency of dosing with alum alone could increase the rates of HBeAg seroconversion [39, 40]. Validation of this observation, however, would require a head-to-head comparison of the effects of the frequency of alum dosing within the same study. Additional strategies that aim to block regulatory or induce stimulatory cytokines should also been considered. IL-10 levels are elevated during chronic disease, and in vitro IL-10 blockade can restore HBV-specific T cell functionality [41]. However, in acute disease, elevated plasma-IL-10 levels have been associated with the attenuation of T cell immunity during acute infection [42] and with hepatic inflammatory flares during acute disease suggesting that in this setting, IL-10 potentially regulates immunopathology. Together, these studies highlight the critical role that IL-10 may play in balancing immunopathology with persistent viremia (reviewed in Couper KN 2008 [43]). Although IL-10 blockade facilitates DNA vaccine-induced T cell responses and enhances clearance of persistent LCMV infection [44], it is unlikely that IL-10 blockade will move into human studies of HBV as an immunotherapeutic or as an adjuvant in the short/medium term, given the risks of immunopathology. IL-12 is also a candidate as this has recently been shown in vitro to enhance the cytotoxicity, polyfunctionality, and multispecificity of HBV-specific T cells and combining IL-12 with blockade of the PD-1 pathway further increased the functionality of CD8+ HBV-specific T cells [45]. Recombinant human IL-12 has been used in cancer immunotherapy, and whilst anti-tumour effects have been limited, IL-12 has been shown to be well tolerated and to induce cellular immunity [46]. Genetic HBV vaccines that encode IL-12 have shown promise as discussed above [32].

## Lessons from HCV immunotherapy-viral vectored technologies

The recent development of viral vectored technology to deliver immunogens has been a major step forward in the field, with the capacity now to generate unprecedented levels of T cells in healthy volunteers. Earlier work from the Merck HIV and Oxford malaria vaccine programs used replicative defective adenoviral vectors to prime T cell responses [47]. However, one of the limitations of this technology is the presence of pre-existing anti-vector immunity that may attenuate vaccine responses. However, the use of chimpanzee adenoviruses, to which humans have no prior exposure, can overcome this issue [48].

Important lessons can be learnt from our recent studies of immunotherapy in HCV infection using chimpanzee adenovirus that may also now be applicable to immunotherapy for HBV. In collaboration with an SME Okairos (Rome, Italy), we have recently shown that vaccination with adenoviral vectors derived from chimpanzee (AdCh3) and rare human adenovirus serotypes (AdHu6) encoding the non-structural (NS) proteins of HCV induce a very high magnitude of durable T cell responses, with a broad specificity in healthy volunteers [49]. The key observations that viral load is inversely correlated with CD8+ T cell function in chronic HBV disease [50], the association of class-II alleles with viral persistence [51], and that CD4+ and CD8+ blocking experiments are associated with viral persistence in chimpanzees [8] show that both T cell subsets will need to be induced during immunotherapy.

Our recent data show that heterologous boosting following adenoviral prime with modified vaccinia Ankara (MVA) encoding the same immunogen further enhances both CD8+ and CD4+ T cell subsets in healthy individuals [49]. This strategy shows real promise as a prophylactic vaccine strategy and is now in efficacy testing in Baltimore in a large population of intravenous drug users at a high risk of exposure to HCV infection (ClinicalTrials.gov NCT01436357).

We have also applied the same vaccine strategy to patients with chronic HCV genotype-1 infection to assess for the first time, the capacity of a potent T cell vaccine to restore adaptive immunity in chronic HCV viremia. Vaccination was performed in the setting of a high HCV viral load, and in the setting of a suppressed viral load 2 or 14 weeks into interferon and ribavirin therapy. Overall, we were able to show that T cells were induced to very high levels in a minority of patients, irrespective of viral suppression [52]. However, in those patients, analysis of the host circulating HCV viral sequence showed significant variation in epitopes targeted by vaccine-induced T cells, in comparison with the vaccine immunogen [53]. Overall, we learnt three important lessons from these studies. Firstly, viral load suppression and vaccination with a demonstrably potent vaccine may not be sufficient to restore adaptive immune responses. Secondly, vaccine-induced T cell responses cannot be interpreted without a detailed knowledge of the sequence of the endogenous virus in vaccinees, and thirdly, it is not possible to assess the potency of a vaccine applied in patients with chronic infection if this vaccine has not been first assessed in healthy volunteers.

Each of these lessons may now usefully be applied to vaccine studies of immunotherapy in HBV infection—though there are a number of important differences. In the HCV immunotherapy studies described above, the assessment of the effect of viral load suppression on T cell restoration is confounded by the fact that interferon (IFN) therapy is known to have immunosuppressive and anti-proliferative effects [54]. A similar study in HBV could use HBV anti-viral suppression free from these effects. Also the viral diversity of HBV is much reduced compared with HCV, so that the capacity of a vaccine to induce T cells that do not cross-react with circulating virus is reduced. Nevertheless, HBV viral sequence analysis should be employed for the meaningful interpretation of vaccine-induced T cell responses. And finally, new HBV vaccines should be assessed in healthy people first so that the potency of the vaccine is established. In the generation of a successful HBV immunotherapeutic vaccine, the design of the HBV immunogen will clearly be critical. Since adenoviral vectors can encode large immunogens (>1,500 amino acids) HBV surface, core and polymerase antigens could be encoded in a first generation HBV immunotherapeutic vaccine (see also the article by Kosinska et al. in this issue).

In the HCV studies, we saw no conclusive evidence of immunopathology in vaccinated subjects. This is not surprising given the fact that vaccine-induced T cells rarely targeted endogenous virus. Furthermore, in HCV, only 4 % of hepatocytes are thought to be infected [55], and severe hepatic inflammation associated with adaptive immunity is rarely observed in natural HCV infection. The same may not be true in HBV where the majority of hepatocytes are infected in chronic disease and where fulminant liver failure is associated with adaptive immune responses during primary infection, during seroconversion events in chronic disease, and during immune reconstitution after reactivation of occult HBV infection. The co-administration of oral anti-virals reducing HBV load should significantly reduce the risks of serious immunopathology as new vectors are given to patients for the first time. Thus, immunotherapeutic strategies for HBV will need to tread a careful line aiming to induce immune events associated with HBV seroconversion without inducing severe hepatic inflammation.

The repeated administration of attenuated poxvirus as viral vectors has been assessed by Transgene (France). MVA viral vectors encoding HCV NS proteins (TG4040) were administered repeatedly (*n* = 6) in patients with chronic HCV infection associated with a transient decline in HCV RNA levels in some people [56]. In a phase-II study, this regimen was associated with early viral clearance, but not a statistically significant increase in sustained virological response [57]. Whilst this approach was able to induce HCV-specific T cells, these studies did not evaluate the capacity of vaccine-induced T cells to target endogenous virus in patients.

## cccDNA eradication

The elimination of nuclear cccDNA is required if HBV is to be cured, and this represents a major challenge for HBV immunotherapy. The issue of HBV cccDNA eradication is not easily addressed since cccDNA infects 40–100 % of hepatocytes and has in most cases a lifespan as long as the hepatocyte. It has long been known that the administration of IFN-α therapy in patients with chronic HBV infection, and particularly those with elevated transaminases at baseline may be associated with cure in a minority of people. HBV cure is defined as HBsAg loss and anti-HBs seroconversion which is believed to indicate eradication or inactivation of cccDNA. Furthermore, there is an increased incidence of cure in these patients that continues for some years after the cessation of therapy. It is evident that innate mechanisms are capable of cccDNA eradication. Supporting studies in animal models of HBV, primary hepatocytes, and human liver needle biopsies have demonstrated that interferon-α and lymphotoxin-β receptor activation may upregulate nuclear deaminases (APOBEC3A and APOBEC3B) mediated by HBV core resulting in cccDNA degradation that prevents HBV reactivation [58].

Recent studies sponsored by Gilead sciences have developed a selective oral TLR-7 agonist (GS-9620) with the aim of curing chronic HBV infection. A study in three chimpanzees with chronic infection showed that this compound was able to suppress viral load by >1 log for some months, associated with the stimulation of interferon-α and interferon stimulated genes, chemokines, and natural killer cells. An increase in hepatocyte apoptosis was associated with a fall in serum HBsAg, HBeAg, and HBV antigen positive hepatocytes. [59] Gilead has since moved into human studies. Three clinical trials were started in 2013 people with chronic hepatitis B. Two of the studies have been completed and results are pending. The third study is on-going (ClinicalTrial.gov NCT01590654).

Studies on HIV that aim to cure patients are hoping to induce reactivation of latently infected cells using histone deacetylases inhibitors (HDACi) to induce viral transcription in latently infected cells in patients on HAART, in conjunction with immunotherapeutic approaches [60]. Similar strategies may be developed for HBV immunotherapy. However, the replication-competent HBV cccDNA is not integrated in the host genome and may not be activated by HDACi, and whilst the integrated forms of HBV DNA may be silenced, these are not responsible for the generation of infectious virus. The success of any approach that sought to eliminate cccDNA would most likely rely on the co-administration of oral HBV anti-virals or HBV viral entry inhibitors targeting HBV receptors [61, 62] to prevent the replenishment of the latently infected viral pool.

## Conclusion

In summary, new therapeutic strategies that aim to induce long-term HBV immune control or provide a complete cure through the elimination of cccDNA will be assessed in phase-I/II clinical studies in the short/medium term. These are likely to proceed cautiously, and in a step-wise fashion in patients without significant liver fibrosis and in patients where viral load is suppressed with oral anti-virals therapy. Highly immunogenic vaccines based on adenoviral and MVA vectors used in prime–boost strategies are now available for clinical studies, and the use of simian adenoviruses overcomes the issue of pre-existing vector immunity. The choice of immunogen will be critical but will to some extent require a “test and see” approach in humans. Nevertheless, it would seem prudent to include polymerase, core, and possibly preS/S antigens in a first generation vaccine. Our own data from immunotherapeutic studies of HCV suggest that potent vaccines alone may not be sufficient to restore the function of T cells that have been exposed to antigen for many years and additional strategies such as the co-administration of checkpoint inhibitors and adjuvants that aim to induce innate immunity will also be required. New platforms in viral vectored technology and molecular approaches mean that immunotherapy for HBV that seeks to control and/or cure HBV replication is now a real possibility.
